# A genomic and bioinformatic-based approach to identify genetic variants for liver cancer across multiple continents

**DOI:** 10.5808/gi.23067

**Published:** 2023-12-29

**Authors:** Muhammad Ma’ruf, Lalu Muhammad Irham, Wirawan Adikusuma, Made Ary Sarasmita, Sabiah Khairi, Barkah Djaka Purwanto, Rockie Chong, Maulida Mazaya, Lalu Muhammad Harmain Siswanto

**Affiliations:** 1 Faculty of Pharmacy, Universitas Ahmad Dahlan, Yogyakarta 55164, Indonesia; 2 Departement of Pharmacy, University of Muhammadiyah Mataram, Mataram 83127, Indonesia; 3 Department of Clinical Pharmacy, College of Pharmacy, Taipei Medical University, Taipei 110, Taiwan; 4 Pharmacy Study Program, Faculty of Science and Mathematics, Udayana University, Bali, Indonesia; 5 School of Nursing, College of Nursing, Taipei Medical University, Taipei 11031, Taiwan; 6 Faculty of Medicine, Universitas Ahmad Dahlan, Yogyakarta 55191, Indonesia; 7 PKU Muhammadiyah Bantul Hospital, Bantul, Yogyakarta 55711, Indonesia; 8 Department of Chemistry and Biochemistry, University of California, Los Angeles, Los Angeles, CA 90095, USA; 9 Research Center for Computing, Research Organization for Electronics and Informatics, National Research and Innovation Agency (BRIN), Cibinong Science Center, Cibinong 16911, Indonesia; 10 Mataram Training Health Center, Indonesia Ministry of Health, Mataram 83237, Indonesia

**Keywords:** bioinformatics, genomic variants, liver neoplasms

## Abstract

Liver cancer is the fourth leading cause of death worldwide. Well-known risk factors include hepatitis B virus and hepatitis C virus, along with exposure to aflatoxins, excessive alcohol consumption, obesity, and type 2 diabetes. Genomic variants play a crucial role in mediating the associations between these risk factors and liver cancer. However, the specific variants involved in this process remain under-explored. This study utilized a bioinformatics approach to identify genetic variants associated with liver cancer from various continents. Single-nucleotide polymorphisms associated with liver cancer were retrieved from the genome-wide association studies catalog. Prioritization was then performed using functional annotation with HaploReg v4.1 and the Ensembl database. The prevalence and allele frequencies of each variant were evaluated using Pearson correlation coefficients. Two variants, rs2294915 and rs2896019, encoded by the *PNPLA3* gene, were found to be highly expressed in the liver tissue, as well as in the skin, cell-cultured fibroblasts, and adipose-subcutaneous tissue, all of which contribute to the risk of liver cancer. We further found that these two SNPs (rs2294915 and rs2896019) were positively correlated with the prevalence rate. Positive associations with the prevalence rate were more frequent in East Asian and African populations. We highlight the utility of this population-specific *PNPLA3* genetic variant for genetic association studies and for the early prognosis and treatment of liver cancer. This study highlights the potential of integrating genomic databases with bioinformatic analysis to identify genetic variations involved in the pathogenesis of liver cancer. The genetic variants investigated in this study are likely to predispose to liver cancer and could affect its progression and aggressiveness. We recommend future research prioritizing the validation of these variations in clinical settings.

## Introduction

Liver cancer, a type of carcinoma, has the highest mortality rate in the world each year [1]. In 2018, there were 841,000 new cases of liver cancer, and the death toll reached 782,000 [2]. The average incidence of liver cancer and the associated mortality rate can be two to three times higher in men, particularly in certain regions of the world. According to the Global Cancer Statistics (GLOBOCAN) in 2020, liver cancer was ranked as the third most deadly cancer, responsible for 8.3% of all cancer-related deaths. In that year, there were 905,000 new cases of liver cancer, with a mortality rate of 830,000 [3]. In Indonesia, liver cancer is the second most common cancer among men, with an incidence rate of 12.4 per 100,000 of the population and an average mortality rate of 7.6 per 100,000 [4].

Factors that contribute to liver cancer include chronic infection with hepatitis B virus and hepatitis C virus, exposure to aflatoxin contamination, alcohol consumption, a history of obesity, type 2 diabetes, and smoking addiction [2]. Villanueva [5] notes that additional risk factors may exacerbate the incidence of liver cancer, including an unhealthy lifestyle, geographic conditions, gender, age, family history of the disease, and the extent of liver damage. Liver cancer is also prevalent in regions with high rates of hepatitis B infection. In these areas, the disease often manifests at a younger age, partly because hepatitis B can be transmitted vertically from mother to child during childbirth [6].

Patients often report symptoms such as fatigue, pain, diarrhea, skin abnormalities, and decreased appetite, all of which can adversely affect their quality of life [7]. Consequently, the detection of disease symptoms in liver cancer can involve examining DNA. Variations in genes may be linked to the progression and pathogenesis of diseases, including liver cancer. The genome-wide association studies (GWAS) Catalog is a resource that employs a bioinformatics approach to document genetic variations. This database contains search results for single-nucleotide polymorphisms (SNPs) and has identified several variants associated with liver fat content, circulating liver enzymes, and the development of non-alcoholic fatty liver disease, as well as genetic markers useful in predicting disease disorders [8].

Genetic identification studies in humans aim to identify inherited genetic risk factors for various conditions, including liver cancer. This study used the GWAS catalog database to map genes from genetic variations across several populations that play an essential role in the pathogenesis of liver cancer. The most significant gene variations based on their function in protein changes were further verified.

## Methods

**Fig. 1.**
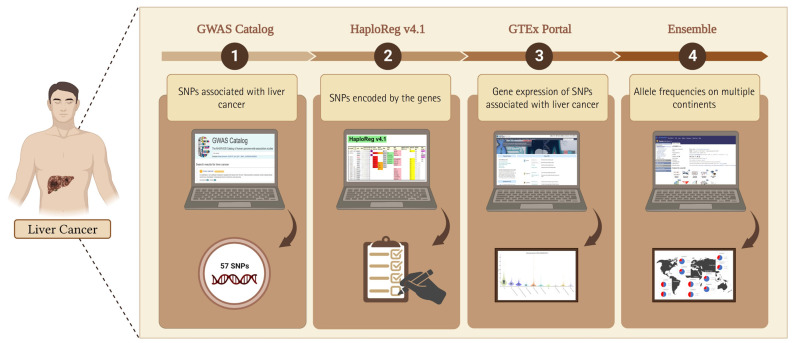
Analytical methodology for integrated bioinformatic, database, and genomic analysis of genetic variations that affect liver cancer. The figure was created with BioRender.com under agreement number “FM25OO073C”. SNP, single-nucleotide polymorphism; GWAS, genome-wide association study.

In this study, we adopted the method used by Ma’ruf et al. [9] and Puspitaningrum et al. [10], as depicted in Fig. 1. Liver cancer-associated SNPs were obtained from the GWAS Catalog database (http://www.ebi.ac.uk/gwas; accessed on 15-02-2023). Subsequently, we performed further analysis using HaploReg (version 4.1) applying a p < 10^-8^ to account for multiple tests in the GWAS catalog. This threshold is commonly used to identify associations between common genetic variants and traits with adjacent gene expression [11]. Furthermore, to evaluate the relationships between various genetic variants and gene expression profiles, we conducted an analysis of expression quantitative trait loci (eQTLs) with data sourced from the GTEx Portal database (http://www.gtexportal.org/home/; accessed on 16 Feb 2023), considering gene expression across various tissues in humans. Additionally, we confirmed the identified variants using the Ensembl Genome Browser (https://www.ensembl.org/index.html; accessed on 17 Feb 2023). Our study considered allele frequencies in populations from Europe, Africa, America, East Asia, and Southeast Asia. To explore the functionalities of different gene variants, we performed evaluations using the SNP nexus database (https://www.snp-nexus.org; accessed on 20 Feb 2023). Furthermore, epidemiological and genomic data on the prevalence of liver cancer rates were obtained from Li et al. [12]. The prevalence rates and allele frequencies of the variants in multiple continents were evaluated using IBM SPSS Statistics 25.0 (IBM Corp., Armonk, NY, USA) with the Pearson correlation test. After the procedure was evaluated, the p-values were obtained. All plots were created using line charts. A p < 0.05 was considered statistically significant in the current study.

## Results and Discussion

### Identification of genomic variants of liver cancer

This study identified SNPs associated with liver cancer from the GWAS catalog. Of these SNPs, 29 were further confirmed through SNP genotyping, as shown in Table 1. Subsequently, HaploReg version 4.1 was utilized, applying a p-value threshold of <10^-8^ based on the number of SNPs obtained. The findings presented in Table 2 indicate an increased risk associated with two genes for liver cancer. The study also analyzed tissue expression impacting liver cancer, with a focus on missense variants of *PNPLA3* (patatin-like phospholipase domain-containing 3).

Through our integrative bioinformatics approach, we prioritized two variants with missense mutations (rs2294915 and rs2896019) that encode the *PNPLA3* gene as biological risk SNPs for liver cancer. Primary liver cancer is a pathological condition characterized by the development of malignant cells within the hepatic tissues. The development of cancer at extraneous anatomical sites that subsequently metastasizes to the liver does not constitute primary liver cancer. Primary liver cancer includes several types, such as hepatocellular carcinoma (HCC), intrahepatic cholangiocarcinoma, and less common varieties like mixed hepatocellular cholangiocarcinoma, fibrolamellar HCC, and the pediatric neoplasm hepatoblastoma [13].

### Gene expression of *PNPLA3* across 10 human tissues

**Fig. 2.**
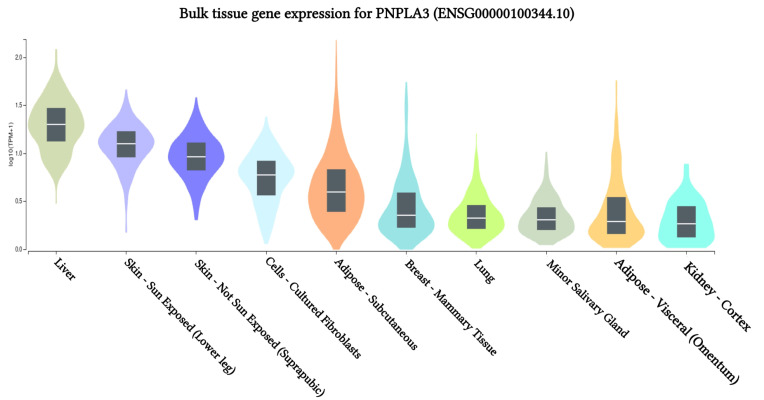
*PNPLA3* gene expression associated with liver cancer across human tissues based on GTEx Portal analysis.

The results of *PNPLA3* gene expression across 10 human tissues revealed significant functional consequences of genetic variation. The highest levels of *PNPLA3* gene expression were observed in the liver, sun-exposed skin (lower legs), non-sun-exposed skin (suprapubic), and adipose-subcutaneous fibroblasts and cell cultures, according to analyses of the 10 human tissues from the GTEx database (Fig. 2). Additionally, we found that the SNP IDs rs2294915 and rs2896019 exhibited similar patterns of gene expression variation in sun-exposed skin (lower legs). Notably, patients with liver cancer often report that their skin appears yellow, which may be related to these findings. Further analysis indicated that the *PNPLA3* gene is also highly expressed in suprapubic and underarm skin.

### Correlation between gene expression of *PNPLA3* and eQTLs

**Fig. 3.**
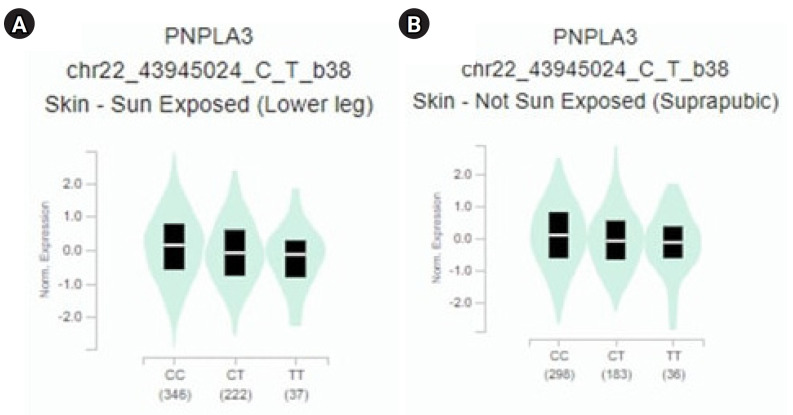
*PNPLA3* gene expression for each genotype of the single-nucleotide polymorphisms: (A) rs2294915 and (B) rs2896019.

The study revealed a correlation between the gene expression of *PNPLA3* and eQTLs. To identify eQTLs associated with liver cancer gene expression, we utilized the GTEx database. We identified minor alleles that are related to liver cancer, as detailed in Table 3 [14]. Notably, we discovered that several SNPs, specifically rs2294915 and rs2896019, exhibit high expression in skin tissue. The CC genotype of both rs2294915 and rs2896019 was associated with increased expression in suprapubic and underarm skin compared to the CT and TT genotypes, as shown in Fig. 3.

The research results show that the genomic database could be used to identify gene variations with significant potential in the pathogenesis of liver cancer. Liver cancer is marked by the yellowing of the eyes and skin [15]. Nessa et al. [16] note that the severity of liver disease can be gauged by the declining quality of liver function. This quality can be evaluated by measuring total bilirubin levels, serum albumin, and prothrombin time.

### Allele frequencies of candidate variants in populations in different continents

**Fig. 4.**
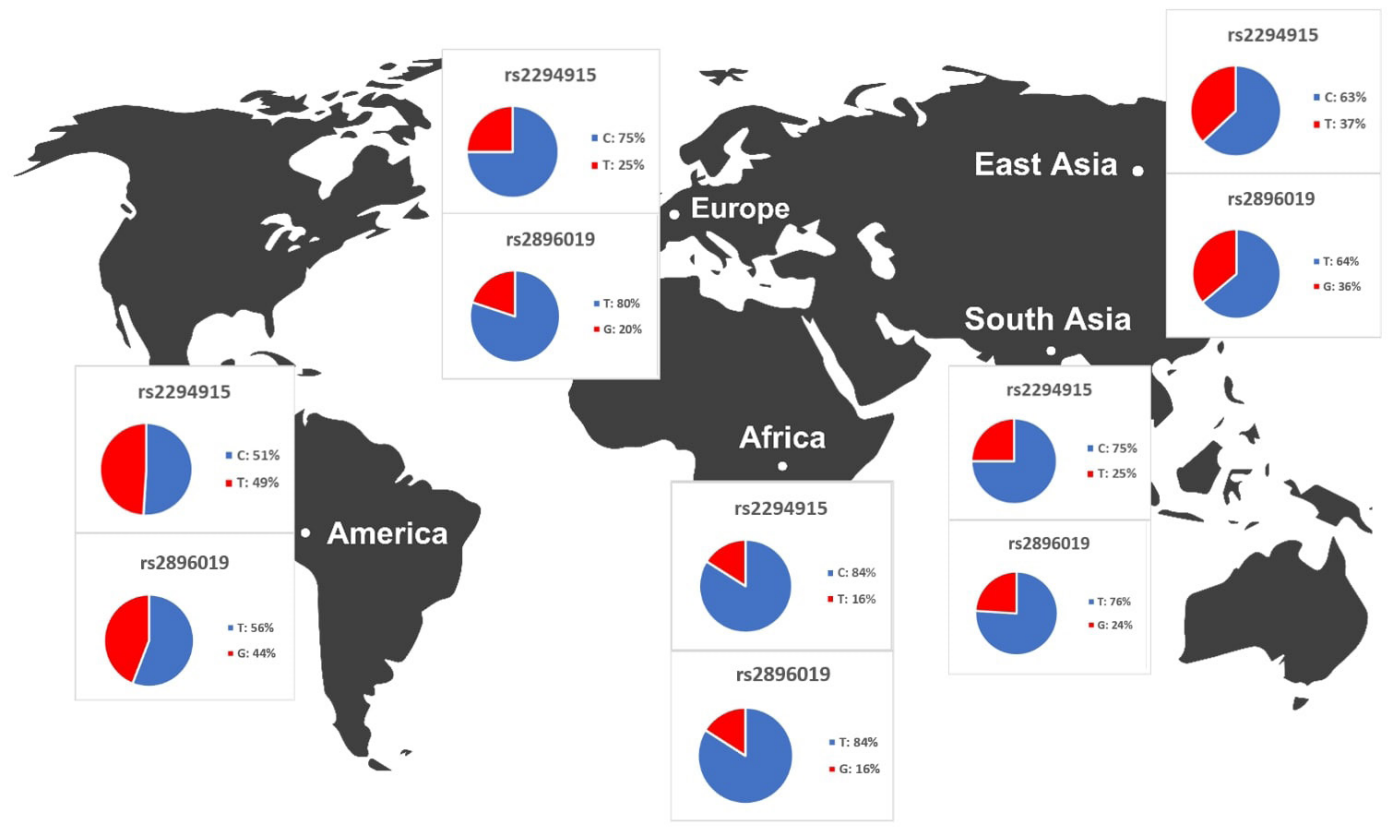
The results of the distribution of *PNPLA3* allele frequencies across various populations.

We identified variants associated with liver cancer gene expression and conducted allele frequency analysis across various populations. As indicated in Table 4, we evaluated the frequency of allele variants in individuals from Europe, America, East Asia, South Asia, and Africa. The allele frequencies for each SNP differed among these populations, as illustrated in Fig. 4. Both Table 4 and Fig. 4 demonstrate that gene expression levels are higher for populations with increased frequencies of the rs2294915 (C) allele and the rs2896019 (T) allele. Specifically, the gene expression associated with the rs2294915 (C) allele was significantly higher in European and South Asian populations compared to those in America, Africa, and East Asia.

Based on these findings, rs2294915 and rs2896019 may be associated with an increased susceptibility to liver cancer, with the highest effect size of -0.50 observed on skin not exposed to sunlight, such as the suprapubic area. Poggiali and Vercelli [17] describe this condition as being characterized by a disruption in the heme biosynthesis pathway, which is due to decreased activity of hepatic uroporphyrinogen decarboxylase. This disruption leads to an accumulation of light-sensitive by-products, including uroporphyrinogen, resulting in the development of skin fragility and blistering in areas exposed to the sun, as well as impaired liver function.

The allele frequencies of the T and G alleles at loci rs2294915 and rs2896019 were significantly lower in African populations compared to those in American, European, and Southeast Asian populations. Overall, the allele frequencies of the variant alleles rs2294915 and rs2896019 suggest they may contribute to the prevalence of variants affecting the gene expression of *PNPLA3*.

**Fig. 5.**
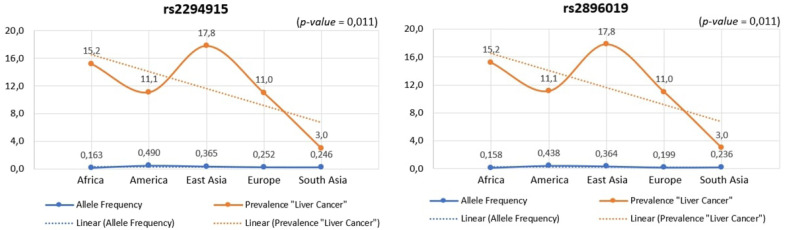
The association between allele frequency and the prevalence of liver cancer on each continent.

Across human populations, the frequency of the T allele at rs2294915 is associated with high expression of *PNPLA3* in liver cancer. This frequency is much lower in African populations (16%) compared to South Asians (25%), Europeans (25%), East Asians (37%), and Americans (49%). Conversely, the frequency of the C allele at rs2296019 is considerably higher in African (84%), European (80%), South Asian (76%), East Asian (64%), and American (56%) populations. Next, we evaluated the association between allele frequency and the prevalence of liver cancer on each continent. Data on liver cancer prevalence were obtained from Li et al. [12,18]. In this context, two SNPs (rs2294915 and rs2896019) were found to be positively correlated with the prevalence rate of liver cancer across multiple continents (Africa, America, East Asia, Europe, South Asia), as determined by Pearson's correlation analysis (p = 0.011) (Fig. 5). Populations with higher frequencies of variant alleles of these polymorphisms are thought to have a higher prevalence of liver cancer. We highlighted that these two variants (rs2294915 and rs2896019) are more frequent in East Asian and African populations, which exhibit higher aggressiveness of liver cancer compared to America, Europe, and South Asia. This study suggests that individuals in East Asian and African populations carrying the variant alleles rs2294915 and rs2896019 may be more susceptible to liver cancer.

Patients with liver cancer who also have a history of alcohol abuse, consuming ≥3 drinks per day, have a 16% increased risk of developing liver cancer compared to the general population. Additionally, individuals with diabetes and those with central obesity are at twice the risk of developing liver cancer [1]. The diagnosis of liver cancer typically involves serological testing combined with imaging techniques, which is the standard approach for detecting liver carcinoma. However, the sensitivity of the commonly used serological test, which is designed to detect alpha-fetoprotein, is only about 60%. Imaging modalities such as magnetic resonance imaging, computed tomography, and ultrasonography demonstrate high levels of sensitivity and specificity in detecting liver cancer, especially in patients with liver cirrhosis [19].

Variant alleles (rs2294915 and rs2896019) are associated with liver cancer. Populations from Africa, America, East Asia, Europe, and South Asia exhibit associated *PNPLA3* expression, which leads to an increased susceptibility to liver cancer. The identification of unique and pathogenic gene variations for a disease is of great interest for both research and clinical validation. These variants provide insights into disease susceptibility and also act as potential diagnostic and prognostic biomarkers [20]. Furthermore, they can aid in the identification of drug target candidates, an approach referred to as genomic-driven drug repurposing [21]. We expect that the discovery of candidate gene variations in *PNPLA3* will facilitate successful clinical validation, potentially establishing it as a promising diagnostic and prognostic biomarker for liver cancer.

It is important to acknowledge that the genetic variants identified in this study as potentially pathogenic are based on preliminary investigations using genomic and bioinformatics databases. While these findings provide crucial insights for future researchers aiming to validate these genetic variants in liver cancer patients, it is important to proceed with caution. We strongly recommend that future research includes additional functional annotations to aid in the prioritization of these pathogenic genetic variants.

This study identified genetic variants that influence liver cancer, highlighting the importance of the *PNPLA3* gene in liver tissue. Consequently, these population groups exhibit varying susceptibilities to liver cancer based on the associated *PNPLA3* expression levels. The observed variations in allele frequencies of the two identified variants, rs2294915 and rs2896019, across populations from Africa, America, East Asia, Europe, and South Asia, significantly impact *PNPLA3* gene expression. Our study also demonstrated that these two SNPs (rs2294915 and rs2896019) were positively correlated with the prevalence rate. The positive association of prevalence rates was more frequently observed in East Asian and African populations. The higher the frequency of the variant alleles of these polymorphisms in a population, the higher the estimated prevalence rates. The variants investigated in this study are likely to predispose individuals to liver cancer and could play a role in its progression and aggressiveness. These findings highlight the critical importance of understanding genomic variations for precision medicine and for designing targeted screening strategies for liver cancer across diverse populations on different continents.

## Figures and Tables

**Table 1.** SNPs from the GWAS catalog with a p < 10^-8^

No.	Variation and risk allele	p-value
1	rs2856723	3 × 10^-43^
2	rs34675408	1 × 10^-32^
3	rs9272105	5 × 10^-22^
4	rs913493	5 × 10^-20^
5	rs2294915	2 × 10^-19^
6	rs17401966	2 × 10^-18^
7	rs3096380	1 × 10^-17^
8	rs9275319	3 × 10^-17^
9	rs584368	2 × 10^-14^
10	rs2596542	4 × 10^-13^
11	rs1110446	9 × 10^-13^
12	rs58489806	3 × 10^-12^
13	rs6078460	2 × 10^-11^
14	rs2523961	6 × 10^-11^
15	rs7574865	2 × 10^-10^
16	rs1110446	3 × 10^-10^
17	rs455804	5 × 10^-10^
18	rs58542926	6 × 10^-10^
19	rs2523961	6 × 10-^10^
20	rs8107030	8 × 10^-10^
21	rs10272859	9 × 10^-10^
22	rs190121281	4 × 10^-9^
23	rs9275572	6 × 10^-9^
24	rs2242652	6 × 10^-9^
25	rs188273166	1 × 10^-8^
26	rs708113	1 × 10^-8^
27	rs2896019	2 × 10^-8^
28	rs17047200	3 × 10^-8^
29	rs541860626	5 × 10^-8^

SNP, single-nucleotide polymorphism; GWAS, genome-wide association study.

**Table 2.** Variants and risk alleles of the prioritized SNPs for liver cancer

Variation and risk alleles	Variants near risk allele (r^2^ > 0.8)	p-value	GENCODE	Type of allele
rs2294915	rs738409	2 × 10^-19^	*PNPLA3*	Missense
rs2896019	rs3761472	2 × 10^-8^	*PNPLA3*	Missense

SNP, single-nucleotide polymorphism.

**Table 3.** Results of eQTLs in liver cancer from the GTEx Portal database

SNP	Gencode ID (ENSG00000-)	Gene symbol	p-value	Effect size	Tissue	Expression level
rs2294915	100344.1	*PNPLA3*	2.8 × 10^-8^	–0.15	Skin - sun exposed (lower leg)	CC > CT > TT
	100344.1	*PNPLA3*	5 × 10^-8^	–0.50	Skin - not sun exposed (suprapubic)	CC > CT > TT
rs2896019	100344.1	*PNPLA3*	6.7 × 10^-11^	–0.19	Skin - sun exposed (lower leg)	CC > CT > TT
	100344.1	*PNPLA3*	2 × 10^-9^	–0.22	Skin - not sun exposed (suprapubic)	CC > CT > TT

Source: expression quantitative trait loci (eQTLs) obtained from the GTEx Portal [14]. eQTL, expression quantitative trait loci; SNP, single-nucleotide polymorphism.

**Table 4.** Analysis of allele frequencies for the *PNPLA3* gene from variant annotation (SNPnexus)

SNP	Gene	Location	Allele		Allele frequency (n)				
			Ref	Eff*	AFR	AMR	EAS	EUR	SAS
rs2294915	*PNPLA3*	Missense	C	T	T: 0.163 (215)	T: 0.490 (340)	T: 0.365 (368)	T: 0.252 (254)	T: 0.246 (241)
rs2896019	*PNPLA3*	Missense	T	G	G: 0.158 (209)	G: 0.438 (304)	G: 0.364 (367)	G: 0.199 (200)	G: 0.236 (231)

SNP, single-nucleotide polymorphism; Ref, reference; Eff, alternate; AFR, Africa; AMR, America; EAS, East Asia; EUR, Europe; SAS, Southeast Asia.
